# Iodine to Remove Seams Arising after a Scald

**Published:** 1853-09

**Authors:** 


*Iodine to Remove Seams arising after a Scald.*—Dr. S. Nicholls, of Longford, Ireland, records a case, in the Dublin Medical Press, for August 17th, 1853, in which the strong tincture of iodine was used with much success to remove very unsightly "seams and puckers," which had appeared after the healing of an extensive scald in a child, aged 18 months.

